# Integrative transcriptomics–metabolomics reveals sphingolipid metabolic reprogramming in traditional Chinese medicine syndrome differentiation of rheumatoid arthritis

**DOI:** 10.3389/fimmu.2026.1892145

**Published:** 2026-09-24

**Authors:** Yao Li, Ying Wu, JinXiang Zeng, Xueyan Guo, Weifeng Lv, JunWei Lin, Kun Lin, Qihong Guo, Rong Su

**Affiliations:** 1 Department of Laboratory Medicine, Foshan Hospital of Traditional Chinese Medicine, Guangzhou University of Chinese Medicine, Foshan, Guangdong, China; 2 The Eighth Clinical Medical College of Guangzhou University of Chinese Medicine, Foshan, Guangdong, China; 3 Foshan Key Laboratory of Chinese Medicine, Foshan Hospital of Traditional Chinese Medicine, Guangzhou University of Chinese Medicine, Foshan, Guangdong, China; 4 Guangdong Provincial Clinical Research Center for Laboratory Medicine, Guangzhou, Guangdong, China; 5 Nephrology and Rheumatology Department, Foshan Hospital of Traditional Chinese Medicine, Guangzhou University of Chinese Medicine, Foshan, China

**Keywords:** C17-sphinganine, metabolomics, rheumatoid arthritis, sgpl1, sphingolipid metabolism, TCM syndrome differentiation, transcriptomics

## Abstract

**Objective:**

To investigate sphingolipid metabolism for differentiating heat-dampness syndrome from kidney-liver deficiency syndrome in Rheumatoid Arthritis (RA), identify metabolic biomarkers, and integrate transcriptomic data to elucidate molecular mechanisms.

**Methods:**

40 RA patients (kidney-liver deficiency syndrome, n=20; heat-dampness syndrome, n=20) were enrolled and serum untargeted metabolomics profiling was performed using ultra-performance liquid chromatography-tandem mass spectrometry. A least absolute shrinkage and selection operator logistic regression diagnostic model was constructed, and its performance was assessed by receiver operating characteristic curve and decision curve analyses. Three independent transcriptomic datasets were downloaded from the Gene Expression Omnibus database (GSE55235: 10 RA synovium, 10 osteoarthritis synovium, and 10 normal synovium; GSE93272: RA whole blood and healthy control whole blood; GSE17755: RA peripheral blood mononuclear cells and healthy control peripheral blood mononuclear cells), and differential expression and tissue-specific regulation of sphingolipid-metabolism-related genes were analyzed. Immune cell infiltration was estimated using marker gene set mean expression scoring, and Spearman correlation analysis was used to construct metabolite-clinical indicator networks.

**Results:**

Sphingosine kinase 1 activity (sphingosine-1-phosphate/C17-sphinganine ratio) was significantly higher in the heat-dampness syndrome group than in the kidney-liver deficiency syndrome group (P = 3.45×10^-6^). C17-sphinganine was significantly negatively correlated with sphingosine-1-phosphate (r = -0.555). C17-sphinganine alone distinguished kidney-liver deficiency syndrome from heat-dampness syndrome with an area under the curve of 0.945 and an accuracy of 95%. Sphingosine-1-phosphate lyase 1 was markedly upregulated in rheumatoid arthritis synovium compared with normal synovium (log2 fold change = 1.124, P = 2.17×10^-4^) and also significantly upregulated in peripheral blood albeit with a smaller effect size (log_2_ fold change = 0.134, P = 8.44×10^-4^), demonstrating tissue-specific magnitude-difference regulation. Sphingosine kinase 1 showed a negative correlation trend with neutrophil infiltration. C8-ceramide-1-phosphate was negatively correlated with C-reactive protein (r = -0.47) and positively correlated with mean corpuscular hemoglobin (r = 0.51).

**Conclusions:**

C17-sphinganine is a core metabolic biomarker for distinguishing kidney-liver deficiency syndrome from heat-dampness syndrome, enabling precise syndrome classification with a single measurement. The tissue-specific magnitude-difference regulation of sphingosine-1-phosphate lyase 1 and the sphingosine kinase 1-neutrophil correlative trend provide novel insights into how sphingolipid metabolism participates in traditional Chinese medicine syndrome differentiation in RA.

## 1 Introduction

Rheumatoid arthritis (RA) is a systemic autoimmune disease characterized by chronic synovial inflammation and progressive joint destruction, with a global prevalence of approximately 0.5%–1% (1–3). Although disease-modifying antirheumatic drugs and biologic agents have markedly improved the prognosis of RA patients, 30%–40% still respond inadequately to current therapies (4, 5), suggesting substantial molecular heterogeneity and an urgent need for more precise individualized treatment strategies (6, 7). Traditional Chinese Medicine (TCM) has a long history in treating RA, with ‘syndrome differentiation’ serving as the cornerstone of individualized therapy (8–10). Heat-dampness syndrome and kidney-liver deficiency syndrome are the two most common TCM syndromes in clinical RA practice (11, 12). Heat-dampness syndrome typically manifests in the active stage of the disease, characterized by joint redness, swelling, heat, pain and elevated inflammatory markers; kidney-liver deficiency syndrome predominantly occurs in the later stages, with dull joint pain, soreness and weakness of the lower back and knees, and anemia tendency (13, 14). Our previous untargeted metabolomics study identified sphingolipid metabolism as a significantly perturbed pathway between these two syndromes (15). Sphingolipid metabolism is an essential network for maintaining cell membrane structure and signal transduction (16–18). Sphingosine-1-phosphate (S1P) is its core signaling molecule, participating in immune cell migration and inflammatory cytokine release through five S1P receptor subtypes (19, 20). S1P is synthesized by sphingosine kinase 1/2 (SPHK1/2) and irreversibly degraded by S1P lyase (SGPL1) (21, 22). Recent studies have confirmed that the S1P signaling pathway plays a critical role in RA synovial inflammation (23–25), and SPHK1 can promote the release of inflammatory cytokines from fibroblast-like synoviocytes (26, 27). With the widespread adoption of high-throughput technologies, public databases such as the Gene Expression Omnibus provide resources for cross-dataset validation (28), and immune infiltration analysis tools enable assessment of tissue immune microenvironments from transcriptomic data (29). Oxidative stress plays an important role in RA pathogenesis (30, 31); glutathione depletion can lead to GPX4 inactivation and thereby trigger ferroptosis (32–34). However, the role of crosstalk between oxidative stress and sphingolipid metabolism in RA syndrome differentiation remains unclear. This study integrates serum metabolomics with multi-dataset transcriptomics to systematically evaluate the diagnostic utility of sphingolipid metabolites for RA syndrome classification and to characterize the tissue-specific regulation of sphingolipid metabolism genes, aiming to provide a molecular basis for the objective diagnosis of TCM syndromes in RA. The study design flowchart is shown in **Figure 1**.

**Figure 1:**
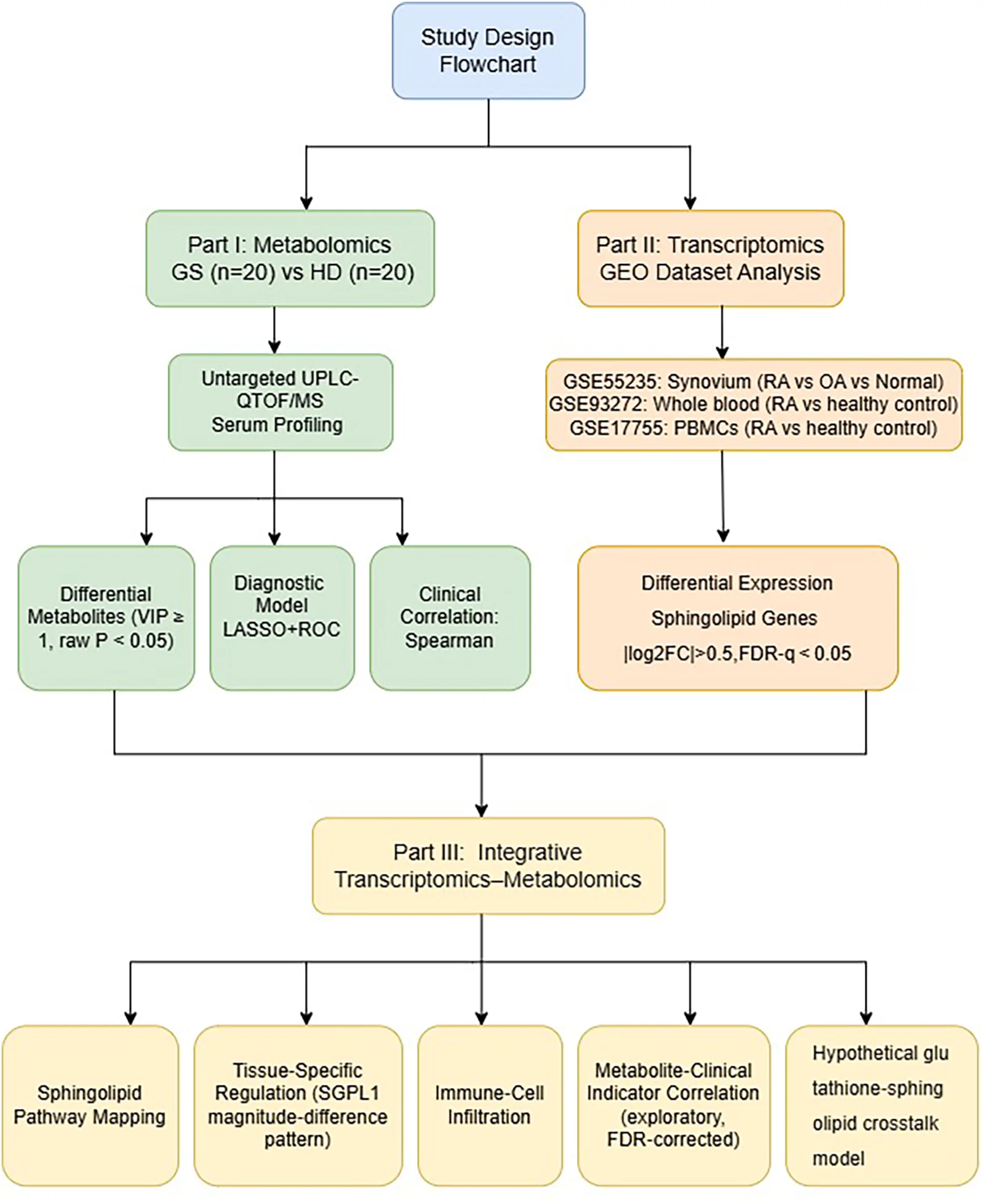
Flow chart of the study design.

## 2 Materials and methods

### 2.1 Study subjects

The metabolomics data were obtained from our previous study (15). Forty RA patients were enrolled and classified into GS (n=20) and HD (n=20) groups according to the Guiding Principles for Clinical Research on New Drugs of Traditional Chinese Medicine. Demographic and clinical data, including age, sex, BMI, disease duration, medication use (methotrexate, glucocorticoids, leflunomide, hydroxychloroquine), and laboratory parameters (CRP, ESR, RF, ACCP, complement C3/C4, liver and renal function tests, and hematological indices), were systematically collected from electronic medical records. Inclusion criteria: fulfillment of the 1987 American College of Rheumatology or the 2010 ACR/EULAR classification criteria for RA. Exclusion criteria: concurrent other autoimmune diseases, severe hepatic or renal insufficiency, or malignant tumors. This study was approved by the Ethics Committee of Foshan Hospital of Traditional Chinese Medicine, and written informed consent was obtained from all subjects.

### 2.2 Metabolomics profiling

Fasting venous blood (5 mL) was collected and centrifuged to obtain serum. Untargeted metabolomics analysis was performed using an Agilent 1290 UPLC system coupled with an Agilent 6545 QTOF mass spectrometer, with data acquired in both positive and negative ion modes. Peak alignment and metabolite identification were performed using MSDIAL (v4.24) with reference to Metlin, MassBank, MoNA, and HMDB databases (35). Differential metabolites were screened using variable importance in projection ≥ 1 in PLS-DA and Student’s t-test P < 0.05. Metabolic pathway enrichment analysis was performed based on the KEGG database (36).

### 2.3 Diagnostic model construction

SPHK1 activity was calculated as the S1P/C17-Sphinganine ratio. A combined diagnostic model was constructed using LASSO logistic regression. Discriminatory ability was evaluated by ROC curve analysis, with calculation of AUC, sensitivity, and specificity. The DeLong test was used to compare AUCs between different models. Decision curve analysis was performed to evaluate clinical net benefit.

### 2.4 Transcriptomic data acquisition and processing

Three datasets were downloaded from the GEO database (28): GSE55235 (10 RA synovium, 10 OA synovium, 10 normal synovium), GSE93272 (RA whole blood an Differential expression analysis was conducted using GEO2R with screening thresholds of |log2FC| > 0.5 and P < 0.05. healthy control whole blood), and GSE17755 (RA PBMCs and healthy control PBMCs). A set of 29 core sphingolipid metabolism genes was defined based on the KEGG pathway.

### 2.5 Immune infiltration analysis

Based on the GSE55235 expression matrix, the relative abundance of immune-cell-signature scores (CD8^+^ T cells, CD4^+^ T cells, B cells, NK cells, macrophages, and neutrophils) was estimated using marker gene set mean expression scoring (29). Immune cell signature scores were calculated as the mean expression of curated marker gene sets: CD8A/CD8B/GZMB/PRF1 for CD8^+^ T cells; CD4/CD3D/CD3E/CD3G for CD4^+^ T cells; CD19/CD79A/CD79B/MS4A1 for B cells; NKG7/GNLY/KLRB1/KLRD1 for NK cells; CD68/CD163/CD14/CSF1R for macrophages; and FCGR3B/CXCR2/CSF3R/S100A8 for neutrophils. For probe-to-gene conversion, multiple probes mapping to the same gene symbol were aggregated by taking the maximum expression value.

### 2.6 Statistical analysis and figure preparation

Statistical analyses were performed using R 4.2.3. Quantitative data are presented as mean ± SD or median (IQR) according to distribution, and categorical variables are presented as n (%). Intergroup comparisons were performed using Student’s t-test or Mann–Whitney U test for continuous variables, and Chi-square test or Fisher’s exact test for categorical variables.

To assess the independent association between sphingolipid metabolites and TCM syndrome, partial correlation analysis (Spearman’s method) was performed between C17-sphinganine and S1P, adjusting for covariates including CRP, ESR, cystatin C, methotrexate use, and glucocorticoid use. Multivariate logistic regression was performed with TCM syndrome (GS vs HD) as the dependent variable, adjusting for CRP, ESR, and methotrexate use, with results reported as odds ratios (OR) and 95% confidence intervals (CI). Decision curve analysis (DCA) was performed to evaluate the clinical net benefit of the C17-Sphinganine-based model across a range of threshold probabilities.

Benjamini–Hochberg (BH) false-discovery-rate (FDR) correction was applied for all large-scale Spearman correlation tests to mitigate multiple-comparison-derived false-positive findings. For metabolite-clinical indicator correlation analyses (52 metabolites × 40 clinical indicators, total 2,080 pairwise tests), FDR-q < 0.05 was defined as the significance threshold.

For immune-gene correlation analyses, Spearman’s rank correlation was performed between six sphingolipid-related genes (SGPL1, SPHK1, SPHK2, CERS4, ASAH1, UGCG) and six immune cell signature scores across combined RA and OA synovial samples (n = 20) from the GSE55235 dataset, yielding a total of 36 pairwise tests. Immune cell signature scores were estimated using marker-gene-set mean-expression scoring. Given the exploratory nature and limited sample size of this synovial tissue cohort, an FDR threshold of q < 0.1 was adopted for suggestive correlation trends. A two-tailed P < 0.05 was considered statistically significant for all remaining statistical analyses.

The hypothetical working-model diagram (**Figure 2**) was generated using the BioGDP online scientific illustration platform.

**Figure 2:**
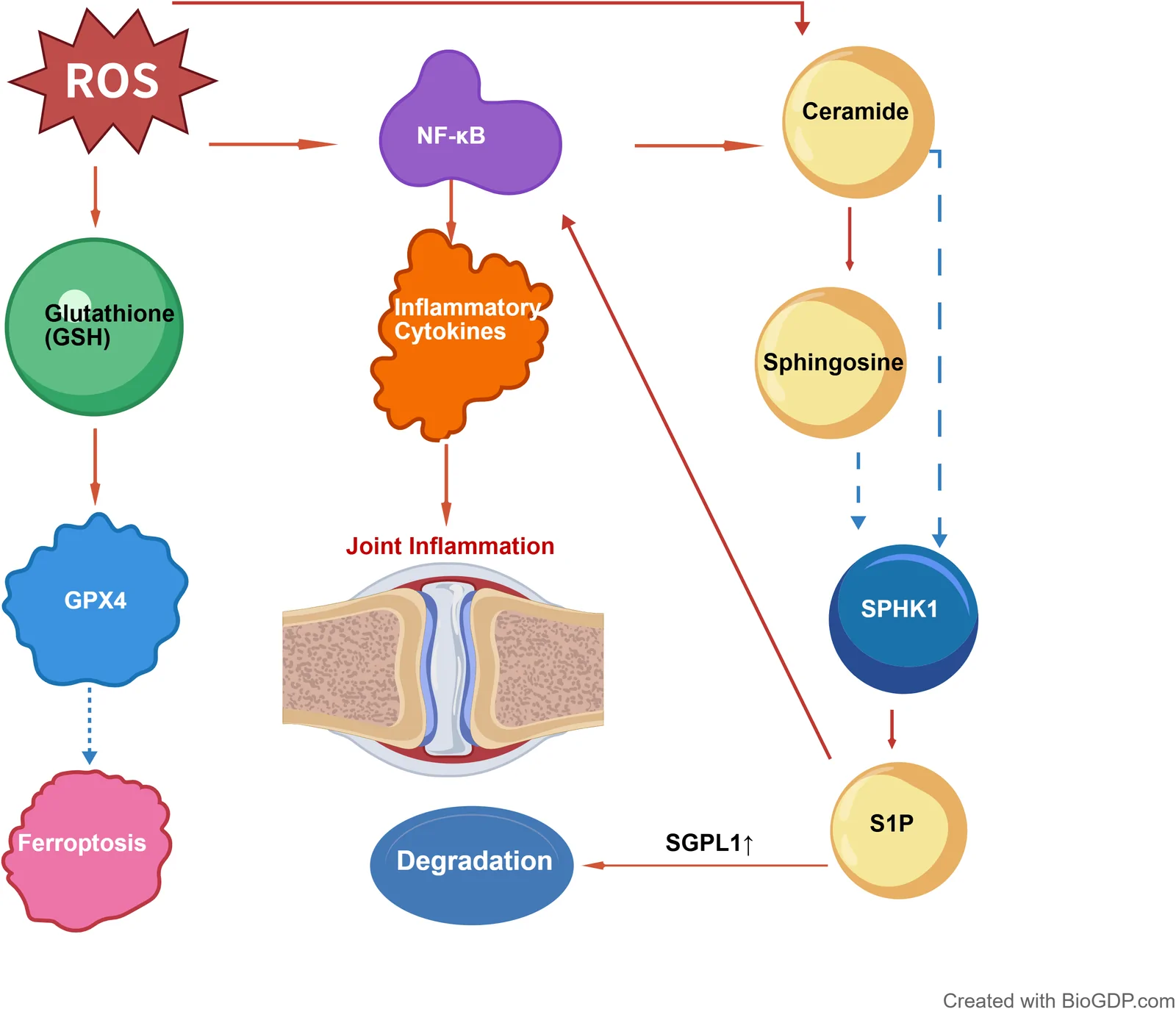
Schematic diagram of the hypothetical working model for glutathione-sphingolipid metabolic crosstalk. The diagram illustrates the crosstalk network between oxidative stress-driven glutathione depletion and sphingolipid metabolic remodeling. Solid red arrows indicate promotion or conversion; dashed blue arrows indicate inhibition. This model is generated from integrative multi-omics correlation analysis and remains to be functionally validated. ROS, reactive oxygen species; GSH, glutathione; GPX4, glutathione peroxidase 4; SPHK1, sphingosine kinase 1; S1P, sphingosine-1-phosphate; NF-κB, nuclear factor kappa B.

## 3 Results

### 3.1 Baseline characteristics of the study population

The baseline clinical and laboratory characteristics of the 40 RA patients stratified by TCM syndrome are summarized in **Table 1** and **Supplementary Table S1**. The GS and HD groups were well-balanced with respect to age, sex, BMI, disease duration, and routine inflammatory markers including CRP and ESR (all P > 0.05). The proportions of patients receiving methotrexate, glucocorticoids, leflunomide, and hydroxychloroquine were also comparable between the two groups (all P > 0.05), excluding medication use as a major confounder. However, significant inter-group differences were observed for cholinesterase (ChE), uric acid, and monocyte percentage (P < 0.05). These findings establish that the two TCM syndrome groups are broadly comparable in demographic and clinical parameters, supporting the validity of subsequent metabolomic comparisons.

**Table 1:** Baseline characteristics and sphingolipid metabolites of ra patients stratified by TCM syndrome.

Variable	HD (n=20)	GS (n=20)	P value
Demographics			
Age, years, mean ± SD	62.50 ± 1.69	63.16 ± 1.66	0.860
BMI, kg/m², mean ± SD	24.01 ± 3.01	23.09 ± 2.98	0.970
Disease duration, years, median (IQR)	3.0 (1.0–6.5)	3.0 (2.0–7.8)	0.586
Sex, Female, n (%)	6 (30%)	4 (20%)	0.715
Inflammatory markers			
CRP, mg/L, mean ± SD	54.00 ± 14.27	56.13 ± 14.94	0.850
ESR, mm/h, mean ± SD	56.26 ± 7.06	57.17 ± 7.54	0.870
RF, IU/mL, mean ± SD	111.7 ± 32.78	115.7 ± 21.29	0.080
ACCP, RU/mL, mean ± SD	63.44 ± 7.92	66.06 ± 7.43	0.640
Sphingolipid metabolites			
C17-Sphinganine, median (IQR)	78,997 (23,751–144,624)	230,303 (222,379–238,844)	<0.001
Sphingosine-1-Phosphate (S1P), median (IQR)	207,893 (27,606–259,534)	24,374 (22,501–27,301)	<0.001
SPHK1-related metabolic ratio (S1P/C17-Sphinganine), median (IQR)	4.66 (0.18–9.29)	0.11 (0.10–0.11)	<0.001
Medications, n (%)			
Leflunomide use	11 (55%)	11 (55%)	1.000
Methotrexate (MTX) use	11 (55%)	12 (60%)	1.000
Glucocorticoids (GC) use	19 (95%)	17 (85%)	0.598
Hydroxychloroquine (HCQ) use	12 (60%)	9 (45%)	0.527

Data are presented as mean ± SD, median (IQR), or n (%). P values were calculated using Student’s t-test, Wilcoxon rank-sum test, or Chi-square/Fisher’s exact test as appropriate. Bold P < 0.05 was considered statistically significant.

HD, heat-dampness syndrome; GS, kidney-liver deficiency syndrome; BMI, body mass index; CRP, C-reactive protein; ESR, erythrocyte sedimentation rate; RF, rheumatoid factor; ACCP, anti-cyclic citrullinated peptide antibody; MTX, methotrexate; GC, glucocorticoids; HCQ, hydroxychloroquine; IQR, interquartile range; SD, standard deviation.

We further investigated whether the discriminative power of these sphingolipid metabolites was driven by their correlation with conventional clinical parameters. Spearman correlation analysis between sphingolipid metabolites and 40 clinical indicators revealed that, after stringent FDR correction (q < 0.05), no metabolite-clinical indicator pair reached statistical significance (**Supplementary Table S2**). The absence of significant correlations with inflammatory markers (CRP, ESR), liver function, renal function, and hematological indices indicates that the sphingolipid metabolic signature is largely independent of routine clinical parameters. This independence reinforces the specificity of C17-Sphinganine as a TCM syndrome-related biomarker, rather than a proxy for systemic inflammation or organ dysfunction, further supporting its potential value for objective syndrome classification.

### 3.2 Diagnostic performance of sphingolipid metabolites for TCM syndrome classification

We first evaluated the potential of three sphingolipid metabolic indices to discriminate between the two TCM syndromes. As shown in **Figure 3A**, C17-Sphinganine was markedly elevated in the GS group compared with the HD group (P = 2.32×10^-4^), whereas S1P(d18:1) showed the opposite pattern, being significantly higher in HD patients (P = 4.19×10^-4^). Consequently, the SPHK1-related metabolic ratio (estimated as the S1P/C17-Sphinganine peak-area ratio, not direct enzymatic activity measurement), was profoundly higher in the HD group than in the GS group (median: 4.66 vs. 0.11, P = 3.45×10^-6^). These reciprocal alterations indicate that GS is characterized by C17-Sphinganine accumulation with suppressed SPHK1 activity, whereas HD is associated with enhanced S1P production and elevated SPHK1 activity.

**Figure 3:**
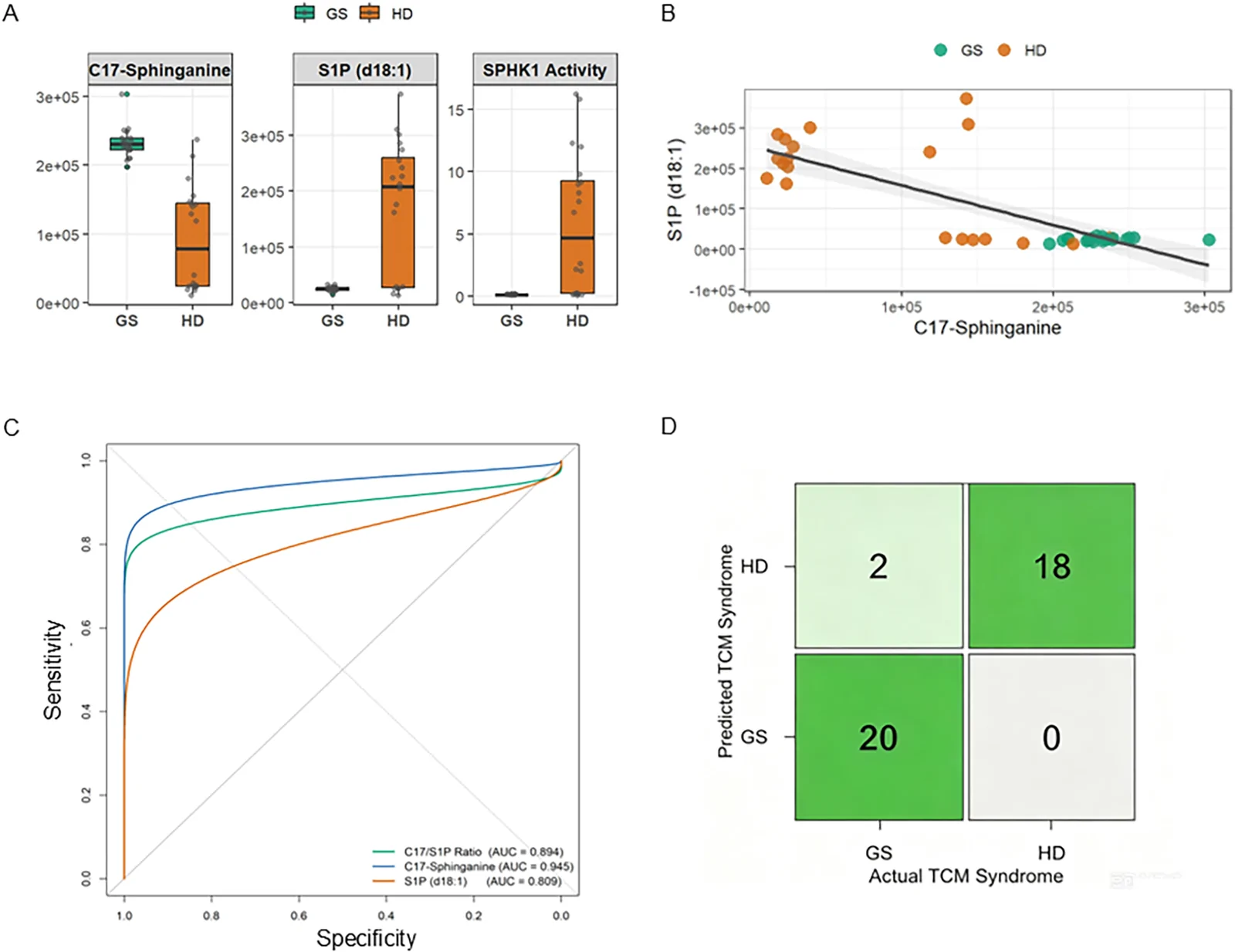
Evaluation of the diagnostic performance of sphingolipid metabolites in distinguishing RA HD from GS. **(A)** Boxplot comparison of C17-Sphinganine, S1P(d18:1), and SPHK1 activity between the GS (n=20) and HD (n=20) groups. Boxes indicate median and interquartile range; whiskers represent data range. Statistical significance was determined by Wilcoxon rank-sum test (***P < 0.001). **(B)** Scatter plot of the correlation between C17-Sphinganine and S1P(d18:1). Green dots represent the GS group; orange dots represent the HD group. **(C)** ROC curves of C17-Sphinganine, S1P(d18:1), and the C17/S1P ratio for distinguishing HD from GS. **(D)** Confusion matrix of the combined logistic regression model. HD, heat-dampness syndrome; GS, kidney-liver deficiency syndrome; S1P, sphingosine-1-phosphate; SPHK1, sphingosine kinase 1; AUC, area under the curve; ROC, receiver operating characteristic.

Spearman correlation analysis revealed a strong inverse relationship between C17-sphinganine and S1P (d18:1) (r = −0.555, P < 0.001, **Figure 3B**). Unlike the high-dimensional metabolite-clinical indicator and gene-immune correlation screens that required rigorous FDR correction, this metabolite-metabolite association represented a pre-specified, hypothesis-driven targeted comparison and did not involve multiple independent statistical tests. Sequential partial correlation analyses further validated this robust inverse association (**Supplementary Table S3**). The significant negative correlation remained stable after stepwise adjustment for conventional inflammatory markers (CRP, ESR), renal indicator (cystatin C), and RA therapeutic regimens (methotrexate and glucocorticoid use). In the fully adjusted model incorporating all above covariates (n = 37), the inverse association persisted steadily (partial r = −0.505, P = 0.0032). These findings demonstrated that the specific reciprocal pattern of C17-sphinganine and S1P is independent of systemic inflammation, renal function status, and clinical medication exposure in RA patients.

ROC curve analysis demonstrated that C17-Sphinganine alone exhibited excellent discriminatory ability (AUC = 0.945), outperforming S1P(d18:1) (AUC = 0.809) and performing comparably to the C17/S1P ratio (AUC = 0.894) (**Figure 3C**). The DeLong test confirmed that C17-Sphinganine was not significantly inferior to the combined ratio (Z = 0.892, P = 0.372). A logistic regression model combining C17-Sphinganine and the C17/S1P ratio correctly classified 20/20 GS patients (100% specificity) and 18/20 HD patients (90% sensitivity), achieving an overall accuracy of 95% (**Figure 3D**).

To further confirm the independent discriminative value of C17-Sphinganine, we performed multivariate logistic regression analysis adjusting for CRP, ESR, and methotrexate use. As shown in **Supplementary Table S4**, C17-Sphinganine remained significantly associated with TCM syndrome (OR = 0.9999, 95% CI: 0.9998–0.99997, P = 0.018), while the covariates were not significant predictors. Collectively, these findings establish C17-Sphinganine as a robust and independent metabolic biomarker for distinguishing the two TCM syndromes in RA.

### 3.3 Decision curve analysis

To evaluate the clinical utility of C17-Sphinganine, we performed decision curve analysis. The results showed that C17-Sphinganine maintained positive net benefit across the full threshold range of 0–1.0 (0.85–1.00), significantly outperforming both the “treat all as HD” and “treat none” extreme strategies (**Figure 4**). As the threshold probability increased, the net benefit of the “treat all” strategy rapidly declined below zero, whereas C17-Sphinganine consistently maintained stable positive net benefit. These findings indicate that, regardless of whether clinicians require a high or low diagnostic certainty, testing C17-Sphinganine to assist TCM syndrome differentiation provides clinical net benefit and has broad practical application value.

**Figure 4:**
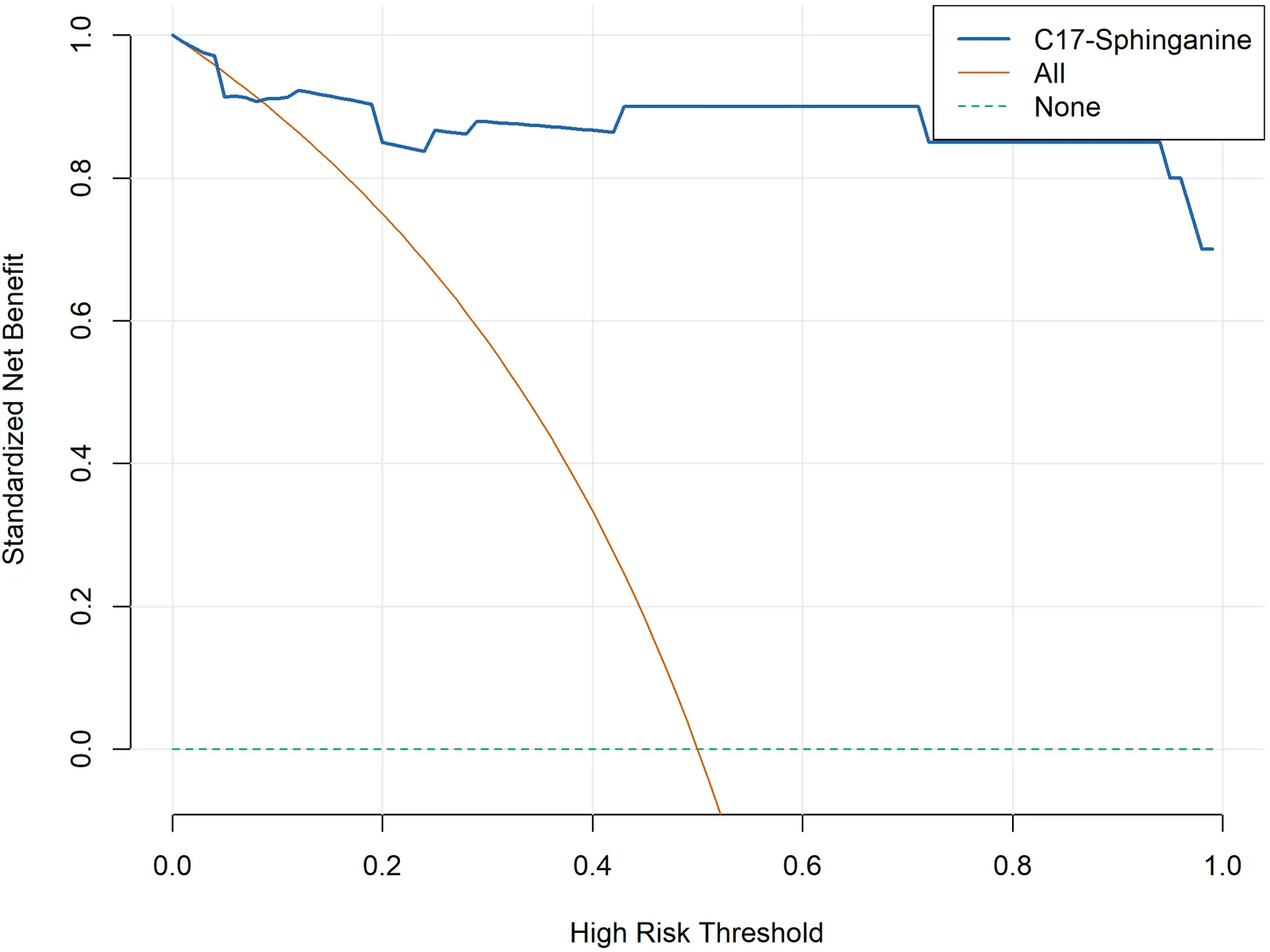
Decision curve analysis of C17-sphinganine for distinguishing GS from HD syndromes. The X-axis represents threshold probability, and the Y-axis represents net benefit. The solid blue line represents the C17-Sphinganine model, the solid red line represents the “treat all as HD” strategy, and the dashed green line represents the “treat none” strategy.

### 3.4 Differential expression of sphingolipid metabolism genes in RA synovium

To characterize the transcriptional landscape of sphingolipid metabolism in the RA joint, we analyzed the GSE55235 dataset comprising RA synovium (n=10), OA synovium (n=10), and normal synovium (n=10). Among a curated panel of sphingolipid metabolic genes, 18 were differentially expressed across the three pairwise comparisons (**Figure 5A**). In the RA versus OA comparison, ASAH1 (log2FC = −1.635, adj.P.Val = 0.041) and CERS4 (log2FC = −1.582, adj.P.Val = 0.017) were the most significantly downregulated genes, while UGCG (log2FC = 1.000, adj.P.Val = 0.035) and SGPL1 (log2FC = 0.636, adj.P.Val = 0.017) were significantly upregulated. SPHK1 showed negligible change (log2FC = 0.053, P = 0.818). In the RA versus Normal comparison, SGPL1 was the most profoundly altered gene (log2FC = 1.124, adj.P.Val = 2.63×10^-6^), with concomitant upregulation of SPTLC2, CERK, and SMPD2 (all log2FC > 0.67, P < 0.015). SPHK1 and SPHK2 showed non-significant downward trends. Conversely, in OA versus Normal, SGPL1 exhibited a diametrically opposite pattern, being significantly downregulated (log2FC = −0.488, adj.P.Val = 0.048), together with CERK (log2FC = −0.657, adj.P.Val = 0.024).

**Figure 5:**
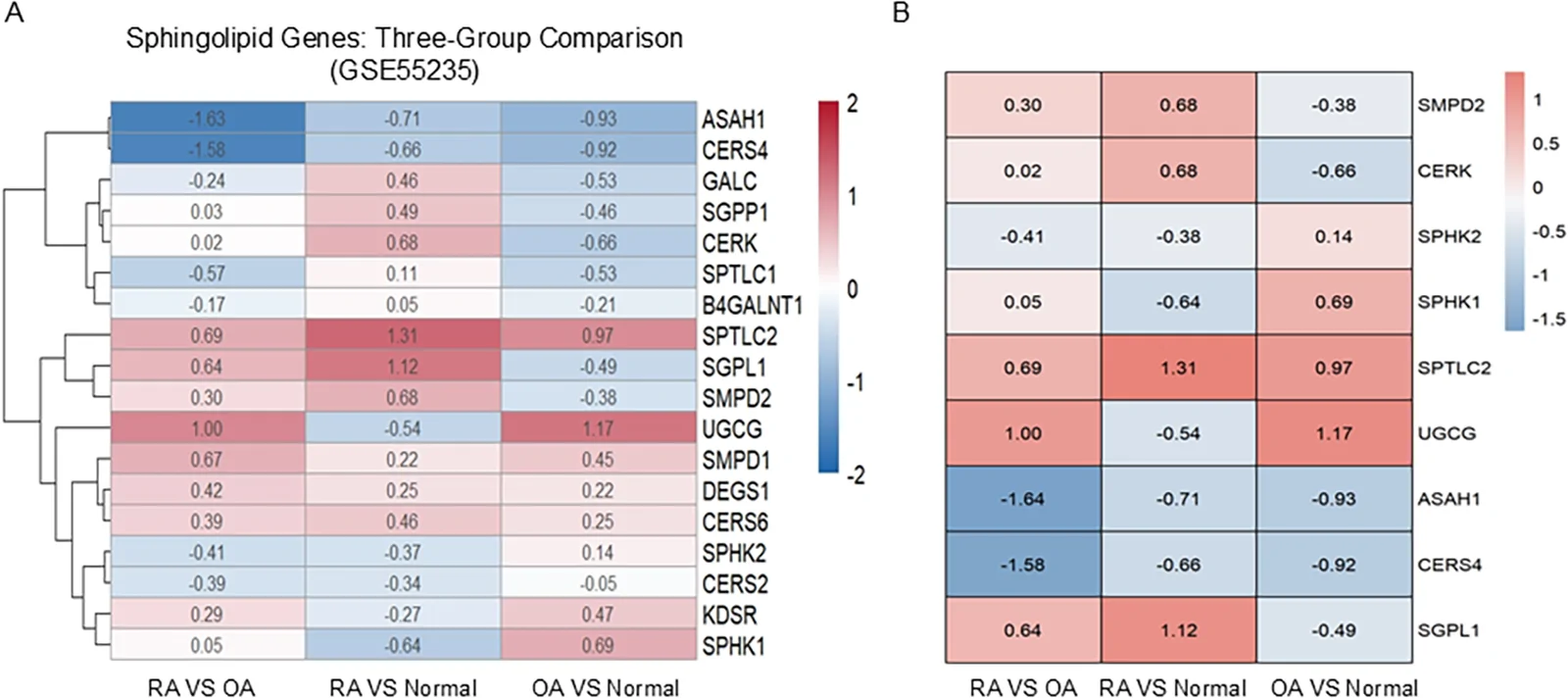
Transcriptomic analysis of sphingolipid metabolism genes in RA synovium. **(A)** Volcano plot of differentially expressed genes between RA and OA synovium in the GSE55235 dataset. Red, significantly upregulated genes (log2FC > 1, adj.P.Val < 0.05); blue, significantly downregulated genes (log2FC < -1, adj.P.Val < 0.05); white, genes with no significant differential expression. **(B)** Heatmap of differential expression of nine key sphingolipid metabolism genes across three comparisons (RA vs OA, RA vs Normal, and OA vs Normal). Red indicates upregulation; blue indicates downregulation. Numerical values represent log2 fold change. P < 0.05, P < 0.01, P < 0.001.

Integrative heatmap analysis highlighted several key patterns (**Figure 5B**). SGPL1 uniquely displayed an RA-specific bidirectional regulatory signature—markedly upregulated in RA yet downregulated in OA. CERS4 and ASAH1 were selectively downregulated in RA versus OA but not in RA versus Normal, suggesting their utility as transcriptional markers for discriminating these two arthritic conditions. SPTLC2 upregulation was common to both RA and OA, indicating generalized enhancement of *de novo* sphingolipid synthesis in synovial pathology. Notably, neither SPHK1 nor SPHK2 showed significant differential expression in any comparison (|log2FC| < 1, P > 0.05), suggesting that S1P synthesis in RA synovium is primarily regulated at the post-translational level or driven by substrate availability rather than transcriptional control of these kinases.

### 3.5 Multi-dataset validation of sphingolipid metabolism gene expression

The above findings were based on synovial tissue. To validate their consistency across different tissue compartments, two additional datasets, GSE93272 (whole blood) and GSE17755 (PBMCs), were included for cross-tissue validation (**Supplementary Table S5**).

SGPL1 expression in peripheral blood showed a pattern partially consistent with that observed in synovium: it was significantly upregulated in GSE93272 (log_2_FC = +0.134, P = 8.44×10^-4^), while no differential expression was identified in GSE17755 (**Figure 6A**). Notably, the effect size of SGPL1 upregulation in GSE93272 was smaller than that observed in the synovial datasets, suggesting a context-dependent magnitude of transcriptional regulation. SPHK1 exhibited no significant differential expression in either peripheral-blood-derived dataset, with near-zero effect sizes in GSE93272 (log_2_FC = +0.084, P = 0.151) and GSE17755 (log_2_FC = +0.018, P = 0.767) (**Figure 6B**). SPHK2 was significantly upregulated in GSE93272 (log_2_FC = +0.176, P = 6.09×10^-6^) but showed no change in GSE17755 (**Figure 6B**). Collectively, these results further suggest that the regulation of S1P synthesis in RA is not consistently driven by transcriptional changes in sphingosine kinases, as SPHK1 and SPHK2 showed negligible or discordant expression alterations across peripheral blood datasets.

**Figure 6:**
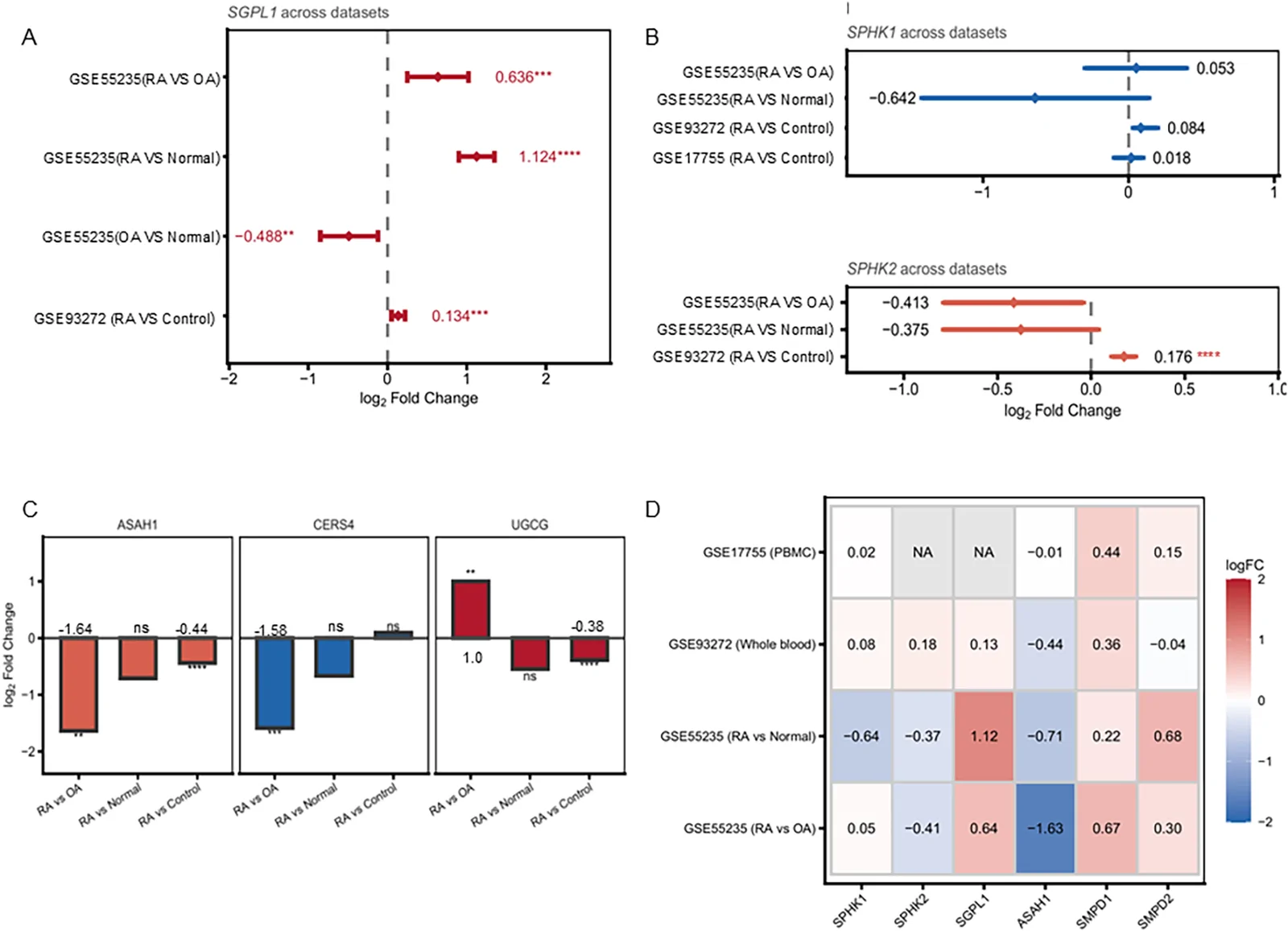
Multi-dataset validation of key sphingolipid metabolism genes. **(A)** Expression of SGPL1 in three synovial comparisons from GSE55235 and in whole blood from GSE93272. SGPL1 was significantly upregulated in both RA synovium and RA whole-blood, albeit with a smaller effect size in peripheral blood. **(B)** Expression of SPHK1 and SPHK2 across four dataset comparisons. Both genes showed small effect sizes across all comparisons, and SPHK2 was significantly upregulated only in whole blood. **(C)** Differential expression of CERS4, ASAH1, and UGCG in GSE55235 and GSE93272. **(D)** Integrated cross-dataset expression heatmap of six sphingolipid metabolism genes. Red, upregulation; blue, downregulation; gray, data not available. ND = Not Detected (not among the differentially expressed gene list). *P < 0.05, **P < 0.01, ***P < 0.001.

CERS4 showed no significant differential expression in GSE93272 (log_2_FC = +0.091, P = 0.189) and was not detected in GSE17755. ASAH1 was significantly downregulated in GSE93272 (log_2_FC = −0.441, P = 1.40×10^-4^), a direction consistent with its downregulation in the RA versus OA synovial comparison, whereas no significant change was detected in GSE17755 (log_2_FC = −0.010, P = 0.809). UGCG was significantly downregulated in GSE93272 (log_2_FC = −0.384, P = 8.65×10^-6^) but was not detected in GSE17755 (**Figure 6C**). The multi-gene integrative heatmap further summarized the expression patterns of all six sphingolipid-related genes across the three datasets, demonstrating that SGPL1 yielded the largest effect size among all tested genes across comparisons, rendering it the most robust differentially expressed gene in our cross-dataset validation (**Figure 6D**).

### 3.6 Integrative transcriptomic-metabolomic analysis of the sphingolipid metabolic pathway

To systematically understand the multi-layered alterations of sphingolipid metabolism in RA synovium, the GSE55235 three-comparison transcriptomic data were mapped onto the KEGG sphingolipid metabolism pathway and integrated with our previous serum metabolomic findings (**Figure 7A**). Pathway analysis revealed bidirectional regulatory characteristics of sphingolipid metabolism in RA synovium. At the *de novo* synthesis end, SPTLC2 was significantly upregulated in RA versus normal synovium (log2FC = 1.31), suggesting activation of the upstream synthetic pathway. In the ceramide metabolic branch, SMPD2 (log2FC = 0.68), ASAH1 (log2FC = 0.42), and CERK (log2FC = 0.68) were all upregulated, promoting sphingomyelin hydrolysis, ceramide degradation, and C1P production, respectively. In the S1P metabolic axis, although SPHK1 (log2FC = -0.64) and SPHK2 (log2FC = -0.38) showed downward trends, SGPL1, which catalyzes S1P degradation, was markedly upregulated (log2FC = 1.12), and SGPP1 was also upregulated (log2FC = 0.49), suggesting that S1P degradation is enhanced in RA synovium. Our previous metabolomic data showed that serum S1P was significantly higher in the HD group than in the GS group, contrasting with the upregulation of degradative enzymes in synovium and suggesting that the joint may accelerate S1P clearance through SGPL1 upregulation to limit local inflammation. Tissue-specific analysis further revealed that SGPL1 was significantly upregulated both in synovium and GSE93272 whole-blood, albeit with a much smaller effect size in peripheral blood (GSE93272, log_2_FC = 0.134, P = 8.44 × 10^-4^) (**Figure 7B**), uncovering a compartmentalized magnitude-difference pattern of sphingolipid-metabolizing gene regulation across tissue compartments in RA.

**Figure 7:**
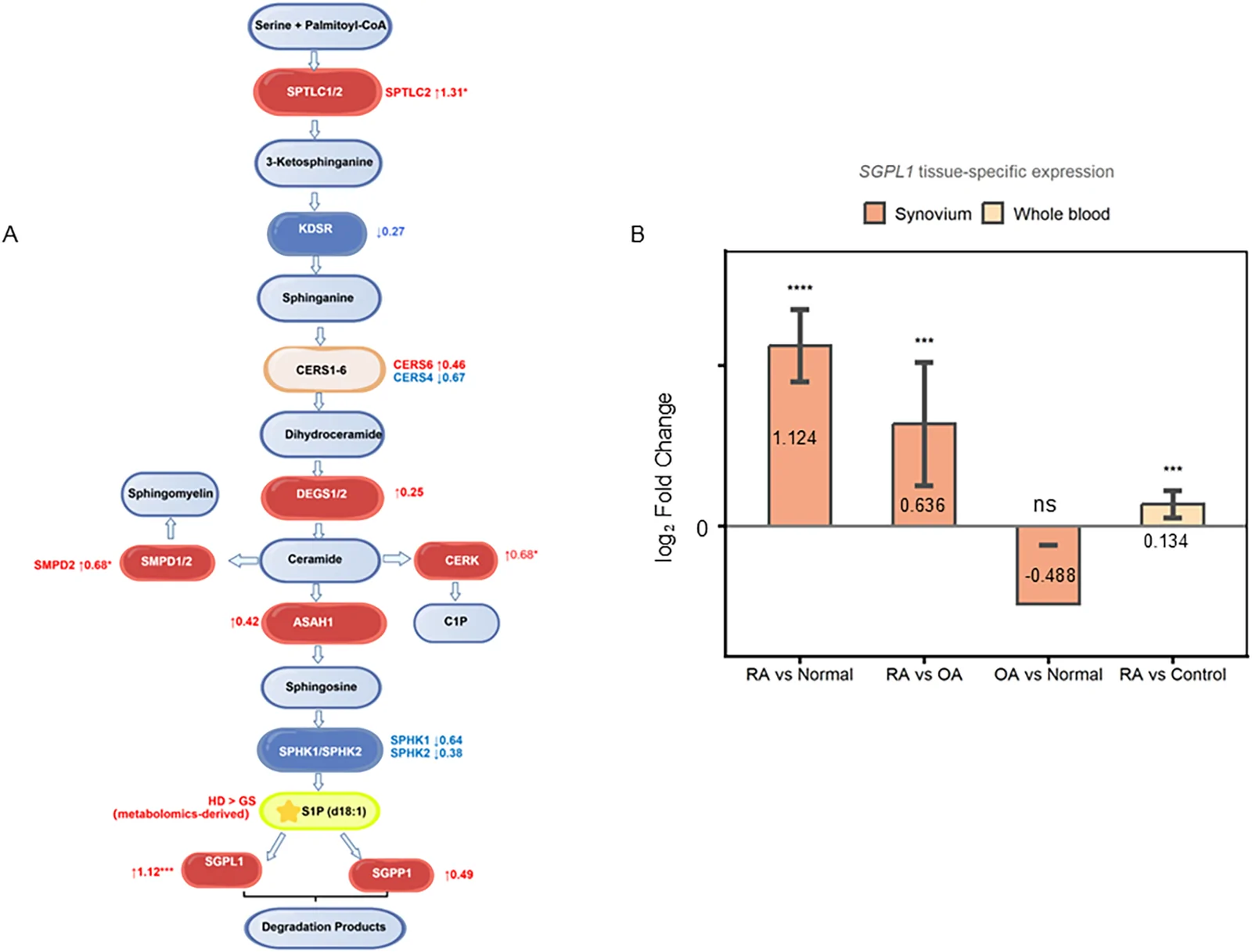
Integrative transcriptomic-metabolomic analysis of the sphingolipid metabolic pathway. **(A)** Schematic diagram of the sphingolipid metabolic pathway. Red text indicates mRNA upregulation; blue text indicates mRNA downregulation (RA vs OA synovium). *P < 0.05, **P < 0.01, ***P < 0.001. **(B)** Tissue-specific expression of SGPL1 in synovium and whole-blood. SGPL1 was significantly upregulated both in synovium and GSE93272 whole-blood, with a smaller effect size observed in peripheral blood.

### 3.7 Immune cell infiltration analysis and correlation with sphingolipid genes

To characterize the immune microenvironment in RA synovium and its association with sphingolipid metabolism, we estimated the relative abundance of six immune-cell signature scores in normal (n = 10), OA (n = 10), and RA synovium (n = 10) from GSE55235 using marker-gene-set mean-expression scoring. Consistent with the box-plot visualization (**Figure 8A**), all six immune-cell signature scores were significantly higher in RA synovium compared with both normal and OA synovium (all P < 0.05, **Supplementary Table S6**). CD8^+^ T cells, CD4^+^ T cells, B cells, and NK cells exhibited approximately 3- to 4-fold higher signature scores in RA relative to normal synovium, while macrophages and neutrophils also showed substantial elevations.

**Figure 8:**
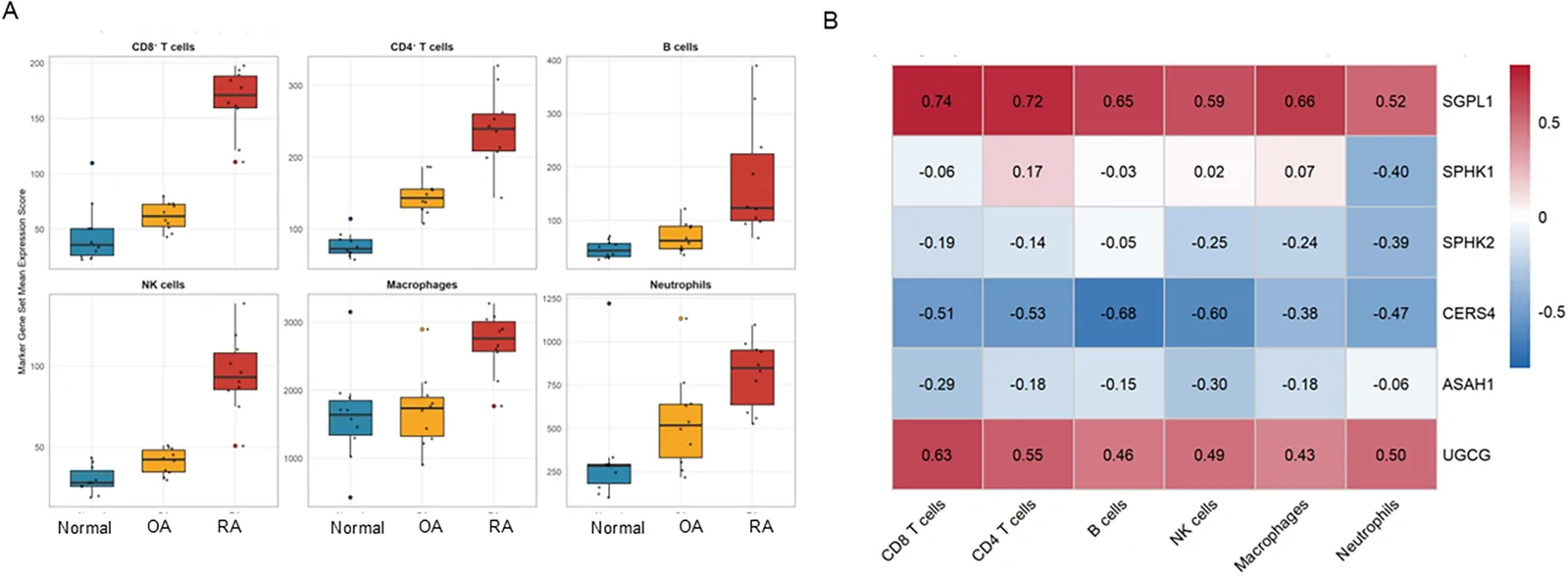
Correlation between sphingolipid-metabolism gene expression and immune-cell signature scores. **(A)** Box-plot comparison of six immune-cell signature scores across normal, OA, and RA synovial samples from the GSE55235 dataset (n=10 per group). Immune-cell relative abundance was estimated by marker-gene-set mean-expression scoring. **(B)** Spearman correlation heatmap between six key sphingolipid-related genes and immune-cell signature scores based on combined RA and OA synovial samples (n=20). Red denotes positive correlation; blue denotes negative correlation. SGPL1, SPHK1, SPHK2, CERS4, ASAH1, and UGCG represent key sphingolipid-metabolism genes identified in **Figure 6**. Full correlation coefficients, raw P-values and FDR-adjusted q-values are provided in **Supplementary Table S7**.

Spearman correlation analysis between six sphingolipid-related genes (SGPL1, SPHK1, SPHK2, CERS4, ASAH1, and UGCG) and immune-cell signature scores was performed on combined RA and OA synovial samples (n = 20) from GSE55235 (**Figure 8B**). After Benjamini-Hochberg FDR correction, associations with FDR q < 0.05 were regarded as statistically significant, whereas 0.05 ≤ q < 0.1 indicated suggestive marginal trends. SGPL1 showed significant positive correlations with CD8^+^ T cells (r = 0.737, q = 0.009), CD4^+^ T cells (r = 0.720, q = 0.009), B cells (r = 0.653, q = 0.017), macrophages (r = 0.657, q = 0.017), and NK cells (r = 0.592, q = 0.031). CERS4 was negatively correlated with B cells (r = −0.679, q = 0.012) and NK cells (r = −0.599, q = 0.027). UGCG positively correlated with CD8^+^ T cells (r = 0.630, q = 0.022) and CD4^+^ T cells (r = 0.555, q = 0.050). Several associations showed marginal trends (0.05 ≤ q < 0.1): SGPL1-neutrophils (r = 0.517, q = 0.063), CERS4-CD8^+^ T cells (r = −0.513, q = 0.063), UGCG-neutrophils (r = 0.496, q = 0.076), UGCG-NK cells (r = 0.489, q = 0.078), CERS4-neutrophils (r = −0.470, q = 0.088), and UGCG-B cells (r = 0.462, q = 0.094). SPHK1 showed a negative trend with neutrophils (r = −0.400, q = 0.161), which was not significant after FDR correction. Most correlations involving SPHK2 and ASAH1 were weak (|r| < 0.40, q > 0.1). Full correlation results are provided in **Supplementary Table S7**.

### 3.8 Glutathione-sphingolipid metabolic crosstalk mechanism

Based on the dual perturbation of glutathione metabolism (the most significantly altered pathway between GS and HD groups) and sphingolipid metabolism (differential pathway between HD and GS) identified in this study, together with the differential expression of SGPL1 from transcriptomic data, we propose an oxidative stress-driven glutathione-sphingolipid metabolic crosstalk model (**Figure 2**). In this model, excessive reactive oxygen species produced locally in the RA joint exacerbate inflammation through two pathways: on the one hand, depleting glutathione and weakening GPX4-mediated suppression of ferroptosis; on the other hand, promoting ceramide generation and driving sphingolipid metabolic flux toward S1P synthesis. S1P in turn activates NF-κB signaling to drive the secretion of pro-inflammatory cytokines, establishing a self-amplifying positive-feedback circuit in which oxidative stress drives sphingolipid remodeling, which in turn amplifies inflammation. This model provides a metabolic mechanistic explanation for the clinical characteristics of HD patients with severe oxidative stress and high inflammation, and also offers a theoretical basis for therapeutic strategies targeting the glutathione-sphingolipid metabolic axis.

## 4 Discussion

By integrating serum metabolomics with multi-dataset transcriptomics, this study provides the first systematic demonstration of the critical role of sphingolipid metabolic reprogramming in TCM syndrome differentiation of RA. The most important finding is that C17-Sphinganine alone efficiently distinguishes GS from HD syndromes (AUC = 0.945, accuracy = 95%), with no significant correlation with CRP or ESR, indicating that this metabolic biomarker reflects metabolic characteristics independent of traditional inflammatory markers.

At the transcriptional level, SGPL1 exhibited the most prominent expression pattern—markedly upregulated in both RA synovium and peripheral blood, though with a smaller effect size in blood. SGPL1 encodes S1P lyase, responsible for irreversible S1P degradation. In classical inflammatory settings, inflammatory stimuli can suppress SGPL1 enzymatic activity, which may occur independent of transcriptional downregulation (37). Conversely, SGPL1-deficient mice exhibit S1P accumulation and IL-23/IL-17/G-CSF axis dysregulation, confirming SGPL1 as a physiological “brake” on S1P-driven inflammation (38). We hypothesize that elevated SGPL1 in RA synovium represents a feedback compensatory response to chronic local S1P signaling. However, this compensation appears insufficient: upstream S1P synthesis (driven by SPHK1-related metabolic activity) may overwhelm degradative capacity, and post-translational mechanisms may limit SGPL1 protein function despite increased mRNA (37).

Recent studies have expanded our understanding of the S1P network in RA. Burg et al. showed that endothelial S1PR1 maintains vascular integrity by limiting VE-cadherin cleavage, and its dysfunction exacerbates arthritis (39). Lee et al. found that S1P2 blockade suppresses Th1/Th17 responses and reduces arthritis severity (40). Chi et al. reported that a herbal extract modulates sphingolipid metabolism to exert anti-RA effects (41). These findings support our proposed model of sphingolipid rheostat imbalance. Furthermore, our crosstalk model resonates with the broader concept of metabolic reprogramming as a central driver of inflammatory polarization in RA, where metabolites act as key regulators of autoimmune diseases beyond mere energy substrates (42, 43). Importantly, TCM may exert therapeutic effects by modulating aberrant metabolic reprogramming to restore immune homeostasis (42). Thus, our findings provide a metabolomic basis for TCM syndrome differentiation and support metabolic intervention as a promising strategy for personalized RA management.

Neither SPHK1 showed significant differential expression in synovium or peripheral-blood datasets. SPHK2 was significantly upregulated only in GSE93272 whole-blood but not in other tested datasets (19–22), suggesting S1P synthesis in RA is primarily regulated post-translationally or by substrate availability. CERS4 and ASAH1 were selectively downregulated in RA versus OA, suggesting they may serve as transcriptional biomarkers for distinguishing RA from OA. In the immune microenvironment, SPHK1 and CERS4 showed negative correlation trends with neutrophils (43–45). Neutrophils are key effector cells in RA synovium, participating in joint destruction through ROS and NET release, and the S1P axis regulates neutrophil chemotaxis and migration (46). SGPL1 showed moderate positive correlations with CD8^+^ and CD4^+^ T cells, suggesting involvement in adaptive immune cell infiltration. However, these correlations are purely associative and require functional validation.

Exploratory Spearman correlation analysis revealed nominal associations between C8-C1P and clinical indicators. Before FDR correction, C8-C1P was tentatively negatively correlated with CRP and positively correlated with MCH; however, no significant correlations remained after multiple-testing correction. Glutathione metabolism, the most significantly altered pathway between GS and HD (15), together with sphingolipid metabolism, constitutes the differential metabolic network. Our hypothetical crosstalk model links oxidative stress with sphingolipid remodeling, providing a potential mechanistic explanation for more severe inflammation in HD patients (32–34).

This study has limitations. Data were derived from different cohorts without sample-level matching, the sample size was small and single-center, SPHK1 activity was indirectly calculated, immune infiltration was estimated by algorithm, and the crosstalk model requires experimental validation. All mechanistic inferences are correlative and cannot establish causality. Single-cell technologies could enable cell-type-specific dissection of SGPL1/SPHK1 expression (46), and proteomic validation is needed to confirm transcriptional-to-protein translation (47).

Future studies should validate the diagnostic threshold of C17-Sphinganine in larger, independent, multi-center RA cohorts. SGPL1 protein-level differences should be verified in paired synovium and blood samples using immunohistochemistry or western blotting. *In vitro* and *In vivo* functional experiments are warranted to clarify the SPHK1-neutrophil correlative trend and validate the proposed crosstalk model. These investigations will be essential to translate our correlative findings into mechanistic understanding and clinical application.

## 5 Conclusions

This study reveals the critical role of sphingolipid metabolic reprogramming in TCM syndrome differentiation of RA. C17-Sphinganine is a core metabolic biomarker for distinguishing GS from HD, enabling precise syndrome classification with a single measurement. The tissue-specific magnitude-difference regulation of SGPL1 and the SPHK1-neutrophil correlative trend provide new perspectives for understanding the molecular mechanisms of RA syndrome differentiation, and the hypothetical glutathione-sphingolipid metabolic crosstalk model offers potential targets for precision diagnosis and treatment of RA.

## Data Availability

The datasets presented in this study can be found in online repositories. The names of the repository/repositories and accession number(s) can be found in the article/**Supplementary Material**.
