# Supplementary material for: Integrative transcriptomics–metabolomics reveals sphingolipid metabolic reprogramming in traditional Chinese medicine syndrome differentiation of rheumatoid arthritis
Source: Front Immunol. 2026 Sep 24;17:1892145. doi: 10.3389/fimmu.2026.1892145 (PMC13646174; doi:10.3389/fimmu.2026.1892145)
Supplement: Supplementary file 1 [file Table1.docx]

**Supplementary tables**

**Supplementary Table S1. Full laboratory parameters of RA patients stratified by TCM syndrome**

| Variable | HD (n=20) | GS (n=20) | P value |
| --- | --- | --- | --- |
| Inflammatory & immune markers |  |  |  |
| ASO, IU/mL, mean ± SD | 55.70 ± 18.80 | 50.68 ± 18.15 | 0.890 |
| Complement C3, g/L, mean ± SD | 1.13 ± 0.06 | 1.00 ± 0.06 | 0.140 |
| Complement C4, g/L, mean ± SD | 0.24 ± 0.03 | 0.27 ± 0.02 | 0.890 |
| IgA, g/L, mean ± SD | 3.09 ± 0.47 | 2.78 ± 0.27 | 0.210 |
| IgG, g/L, mean ± SD | 13.63 ± 1.04 | 14.38 ± 1.18 | 0.450 |
| IgM, g/L, mean ± SD | 1.13 ± 0.19 | 1.26 ± 0.12 | 0.610 |
| Liver & renal function |  |  |  |
| ALT, U/L, mean ± SD | 16.56 ± 3.58 | 14.09 ± 1.29 | 0.510 |
| AST, U/L, mean ± SD | 20.76 ± 3.89 | 17.26 ± 0.76 | 0.370 |
| ALP, U/L, mean ± SD | — | — | — |
| ChE, U/L, mean ± SD | 6.55 ± 0.46 | 10.43 ± 3.40 | <0.001 |
| Creatinine, μmol/L, mean ± SD | 66.69 ± 5.77 | 75.35 ± 8.31 | 0.097 |
| Uric acid, μmol/L, mean ± SD | 278.2 ± 14.67 | 334.4 ± 26.13 | 0.014 |
| Cystatin C, mg/L, median (IQR) | 0.98 (0.84–1.24) | 1.11 (0.93–1.42) | 0.279 |
| Lipid profile |  |  |  |
| Cholesterol, mmol/L, mean ± SD | 4.616 ± 0.23 | 4.603 ± 0.31 | 0.210 |
| Triglycerides, mmol/L, mean ± SD | 1.096 ± 0.11 | 1.118 ± 0.11 | 0.900 |
| Hematology |  |  |  |
| RBC, ×10⁶/L, mean ± SD | 3.68 ± 0.16 | 3.87 ± 0.19 | 0.560 |
| Hemoglobin, g/L, mean ± SD | 106.8 ± 3.29 | 106.5 ± 4.29 | 0.290 |
| MCV, fL, mean ± SD | 85.42 ± 2.12 | 85.62 ± 2.45 | 0.560 |
| MCH, pg, mean ± SD | 26.82 ± 0.80 | 26.67 ± 1.72 | 0.120 |
| MCHC, g/L, mean ± SD | 325.1 ± 2.53 | 331.0 ± 2.36 | 0.790 |
| Lymphocytes, %, mean ± SD | 16.96 ± 1.88 | 20.47 ± 2.29 | 0.440 |
| Monocytes, %, mean ± SD | 5.02 ± 0.51 | 7.23 ± 1.62 | <0.001 |

Data are presented as mean ± SD or median (IQR). P values were calculated using Student’s t-test or Wilcoxon rank-sum test as appropriate. Bold P < 0.05 was considered statistically significant.

Abbreviations: Ig, immunoglobulin; ASO, anti-streptolysin O; ALT, alanine aminotransferase; AST, aspartate aminotransferase; ChE, cholinesterase; RBC, red blood cell; MCV, mean corpuscular volume; MCH, mean corpuscular hemoglobin; MCHC, mean corpuscular hemoglobin concentration; IQR, interquartile range; SD, standard deviation.

**Table S2. FDR-Corrected Spearman Correlations Between Sphingolipid Metabolites and Clinical Indicators**

| Comparison | Total Tests | FDR Threshold (q) | Significant Findings |
| --- | --- | --- | --- |
| 12 metabolites × 40 clinical indicators | 480 | < 0.05 | 0 (None) |

Note: The full 12 × 40 correlation matrix, including all raw correlation coefficients, raw P-values, and FDR-adjusted q-values, is provided as a separate CSV file (Supplementary Table S1_full.csv).

Abbreviations: FDR, false discovery rate; q, FDR-adjusted P-value.

**Table S3. Partial Correlation Analysis Between C17-Sphinganine and S1P**

| Model |  | Adjusted variables | n | Partial r | P value |
| --- | --- | --- | --- | --- | --- |
| Model 1 |  | None (unadjusted) | 40 | -0.555 | <0.001 |
| Model 2 |  | CRP | 37 | -0.548 | <0.001 |
| Model 3 |  | CRP + ESR + Cystatin C | 37 | — | — |
| Model 4 |  | CRP + ESR + Cystatin C + MTX + GC | 37 | -0.505 | 0.0032 |

MTX, methotrexate; GC, glucocorticoids.

**Table S4. Multivariate Logistic Regression Analysis of Factors Associated with TCM Syndrome (GS vs HD)**

| Variable | β coefficient | SE | OR | 95% CI | P value |
| --- | --- | --- | --- | --- | --- |
| C17-Sphinganine | −8.13 × 10⁻⁵ | 3.45 × 10⁻⁵ | 0.9999 | 0.9998 – 0.99997 | 0.018 |
| CRP (mg/L) | 8.39 × 10⁻⁵ | 2.30 × 10⁻² | 1.0001 | 0.9498 – 1.0530 | 0.997 |
| ESR (mm/h) | −3.52 × 10⁻² | 3.61 × 10⁻² | 0.9654 | 0.8785 – 1.0295 | 0.329 |
| Methotrexate use (Yes vs No) | −3.54 × 10⁻² | 1.52 | 0.9652 | 0.040 – 26.78 | 0.981 |

Dependent variable: TCM syndrome (GS = 0, HD = 1; reference: GS).
Model adjusted for: CRP, ESR, and methotrexate use.
n = 37 (3 observations were excluded due to missing values in covariates).
Statistical method: Multivariate logistic regression (binomial family).
Model fit: Null deviance = 51.27 on 36 df; Residual deviance = 12.84 on 32 df; AIC = 22.84.

Data are presented as β coefficient, standard error (SE) , odds ratio (OR) , 95% confidence interval (CI), and P value. Bold values indicate statistical significance (P < 0.05). Abbreviations: GS, kidney-liver deficiency syndrome; HD, heat-dampness syndrome; CRP, C-reactive protein; ESR, erythrocyte sedimentation rate; MTX, methotrexate; OR, odds ratio; CI, confidence interval; SE, standard error; df, degrees of freedom; AIC, Akaike information criterion.

**Table S5. Differential expression of sphingolipid metabolism-related genes across datasets**

| **Gene** | **Dataset / Tissue** | **Comparison** | **logFC** | **Significance** |
| --- | --- | --- | --- | --- |
| *SGPL1* | GSE55235 / Synovium | RA vs OA | 0.636 | *** |
|  |  | RA vs Normal | 1.124 | **** |
|  |  | OA vs Normal | −0.488 | ** |
|  | GSE93272 / Whole blood | RA vs Control | 0.134 | *** |
| *SPHK1* | GSE55235 / Synovium | RA vs OA | 0.053 | ns |
|  |  | RA vs Normal | −0.642 | ns |
|  |  | OA vs Normal | 0.695 | ns |
|  | GSE93272 / Whole blood | RA vs Control | 0.084 | ns |
|  | GSE17755 / PBMC | RA vs Control | 0.018 | ns |
| *SPHK2* | GSE55235 / Synovium | RA vs OA | −0.413 | ns |
|  |  | RA vs Normal | −0.375 | ns |
|  |  | OA vs Normal | 0.136 | ns |
|  | GSE93272 / Whole blood | RA vs Control | 0.176 | **** |
| *CERS4* | GSE55235 / Synovium | RA vs OA | −1.582 | *** |
|  |  | RA vs Normal | −0.665 | ns |
|  |  | OA vs Normal | −0.917 | ns |
|  | GSE93272 / Whole blood | RA vs Control | 0.091 | ns |
| *ASAH1* | GSE55235 / Synovium | RA vs OA | −1.635 | ** |
|  |  | RA vs Normal | −0.708 | ns |
|  |  | OA vs Normal | −0.927 | ns |
|  | GSE93272 / Whole blood | RA vs Control | −0.441 | **** |
|  | GSE17755 / PBMC | RA vs Control | −0.010 | ns |
| *UGCG* | GSE55235 / Synovium | RA vs OA | 1.000 | ** |
|  |  | RA vs Normal | −0.544 | ns |
|  |  | OA vs Normal | 1.167 | ** |
|  | GSE93272 / Whole blood | RA vs Control | −0.384 | **** |

**Abbreviations:** RA, rheumatoid arthritis; OA, osteoarthritis; PBMC, peripheral blood mononuclear cells; logFC, log2 fold change; ns, not significant.

**Significance levels:** **** p<0.0001; *** p<0.001; ** p<0.01; ns, p≥0.05.
Positive logFC values indicate upregulation in the experimental group relative to the comparator; negative values indicate downregulation.

Genes not detected in GSE17755 (SGPL1, SPHK2, CERS4, UGCG) are not shown in this table.

All data were derived from publicly available GEO datasets (GSE55235, GSE93272, GSE17755).

**Table S6. Immune cell infiltration scores in synovial tissues from GSE55235**

| Cell Type | Normal (n=10) | OA (n=10) | RA (n=10) | P (RA vs Normal) | P (RA vs OA) |
| --- | --- | --- | --- | --- | --- |
| CD8 T cells | 45.5 ± 27.5 | 61.4 ± 12.7 | 165.8 ± 29.7 | 0.0002 | < 0.0001 |
| CD4 T cells | 77.5 ± 16.9 | 146.4 ± 25.6 | 239.2 ± 53.5 | < 0.0001 | 0.0002 |
| B cells | 45.5 ± 15.8 | 68.8 ± 27.7 | 174.9 ± 109.6 | < 0.0001 | 0.0007 |
| NK cells | 29.8 ± 8.3 | 41.1 ± 7.9 | 95.4 ± 24.3 | < 0.0001 | < 0.0001 |
| Macrophages | 1618.4 ± 704.1 | 1703.1 ± 554.8 | 2687.3 ± 457.2 | 0.0021 | 0.0011 |
| Neutrophils | 333.3 ± 321.6 | 538.9 ± 274.9 | 812.5 ± 197.1 | 0.0015 | 0.0232 |

Data are presented as mean ± SD of marker gene expression scores. P values calculated by Wilcoxon rank-sum test.

Immune cell infiltration scores in normal, OA, and RA synovial tissues. Immune cell abundance was estimated using gene set mean expression scores of lineage-specific markers from the GSE55235 dataset. RA synovium showed significantly higher infiltration of all six immune cell types compared with both normal and OA synovium. OA, osteoarthritis; RA, rheumatoid arthritis.

**Table S7.** **Full Spearman correlation results between six sphingolipid‑related genes and immune‑cell signature scores in GSE55235 synovial samples**

| **Gene** | **Immune_Cell** | **Correlation (r)** | **P_raw** | **FDR_q** |
| --- | --- | --- | --- | --- |
| SGPL1 | CD8 T cells | 0.73684211 | 0.00031664 | 0.00899589 |
| SGPL1 | CD4 T cells | 0.72030075 | 0.00049977 | 0.00899589 |
| CERS4 | B cells | -0.67920280 | 0.00098998 | 0.01187971 |
| SGPL1 | B cells | 0.65263158 | 0.00232893 | 0.01676827 |
| SGPL1 | Macrophages | 0.65714286 | 0.00212785 | 0.01676827 |
| UGCG | CD8 T cells | 0.63007519 | 0.00358436 | 0.02150617 |
| CERS4 | NK cells | -0.59872140 | 0.00528424 | 0.02717607 |
| SGPL1 | NK cells | 0.59248120 | 0.00688833 | 0.03099750 |
| UGCG | CD4 T cells | 0.55488722 | 0.01236788 | 0.04947152 |
| CERS4 | CD4 T cells | -0.53253110 | 0.01563668 | 0.05629206 |
| SGPL1 | Neutrophils | 0.51729323 | 0.02097838 | 0.06293514 |
| CERS4 | CD8 T cells | -0.51297480 | 0.02072370 | 0.06293514 |
| UGCG | Neutrophils | 0.49624060 | 0.02759330 | 0.07641222 |
| UGCG | NK cells | 0.48872180 | 0.03032449 | 0.07797726 |
| CERS4 | Neutrophils | -0.47010160 | 0.03647422 | 0.08753813 |
| UGCG | B cells | 0.46165414 | 0.04198754 | 0.09447196 |
| UGCG | Macrophages | 0.42556391 | 0.06272233 | 0.13282376 |
| SPHK1 | Neutrophils | -0.40015050 | 0.08042943 | 0.16085887 |
| SPHK2 | Neutrophils | -0.39398500 | 0.08666067 | 0.16419917 |
| CERS4 | Macrophages | -0.37758560 | 0.10073465 | 0.18132238 |
| ASAH1 | NK cells | -0.29774440 | 0.20175953 | 0.34587348 |
| ASAH1 | CD8 T cells | -0.29022560 | 0.21380904 | 0.34986934 |
| SPHK2 | NK cells | -0.24962410 | 0.28720312 | 0.44953532 |
| SPHK2 | Macrophages | -0.23609020 | 0.31482815 | 0.47224223 |
| SPHK2 | CD8 T cells | -0.18646620 | 0.42948009 | 0.59301378 |
| ASAH1 | CD4 T cells | -0.18496240 | 0.43327306 | 0.59301378 |
| ASAH1 | Macrophages | -0.18045110 | 0.44476034 | 0.59301378 |
| SPHK1 | CD4 T cells | 0.16848440 | 0.47766068 | 0.61413516 |
| ASAH1 | B cells | -0.15037590 | 0.52535073 | 0.65215953 |
| SPHK2 | CD4 T cells | -0.13834590 | 0.55943308 | 0.67131969 |
| SPHK1 | CD8 T cells | -0.06468600 | 0.78643634 | 0.86375346 |
| SPHK1 | Macrophages | 0.07295976 | 0.75984516 | 0.86375346 |
| ASAH1 | Neutrophils | -0.06315790 | 0.79177400 | 0.86375346 |
| SPHK2 | B cells | -0.04661650 | 0.84620167 | 0.89597824 |
| SPHK1 | B cells | -0.02783000 | 0.90728190 | 0.93320424 |
| SPHK1 | NK cells | 0.01805190 | 0.93978660 | 0.93978660 |
